# Supplementary material for: Quantitative modeling predicts mechanistic links between pre-treatment microbiome composition and metronidazole efficacy in bacterial vaginosis
Source: Nat Commun. 2020 Dec 1;11:6147. doi: 10.1038/s41467-020-19880-w (PMC7708644; doi:10.1038/s41467-020-19880-w)
Supplement: Supplementary file 1 — Supplementary Information [file 41467_2020_19880_MOESM1_ESM.pdf]

Supplementary Information

Quantitative modeling predicts mechanistic links between pre-treatment microbiome composition and metronidazole efficacy in bacterial vaginosis

Supplementary Figures

Supplementary Figure 1: Kinetic data for parameterization of MNZ interactions with L. iners and G. vaginalis.

Supplementary Figure 2: Model parameterization of growth dynamics with and without MNZ.

Supplementary Figure 3: L. iners 1D sensitivity analysis.

Supplementary Figure 4: Comparison of model predictions with observed levels of MNZ and metabolites.

Supplementary Figure 5: L. iners susceptibility to MNZ does not influence Gv:Li ratio dependent MNZ efficacy.

Supplementary Figure 6: BV:LB ratios influence endpoint BV-associated bacteria and Lactobacillus spp. abundances even with strain variation.

Supplementary Figure 7: Uptake or Sequestration of Lactobacillus spp. with MNZ drives initial BV:LB ratio influence of MNZ efficacy.

Supplementary Figure 8: Initial relative abundance data for L. iners and G. vaginalis for clinical outcomes.

Supplementary Tables

Supplementary Table 1: Parameters for two species model.

Supplementary Table 2: Parameter ranges used to simulate intra-species variability.

Supplementary Table 3: Inter-species interaction terms.

Supplementary Table 4: UMB-HMP cohort data.

Supplementary Table 5: CONRAD BV cohort data.

Supplementary Equations

Base Model Equations

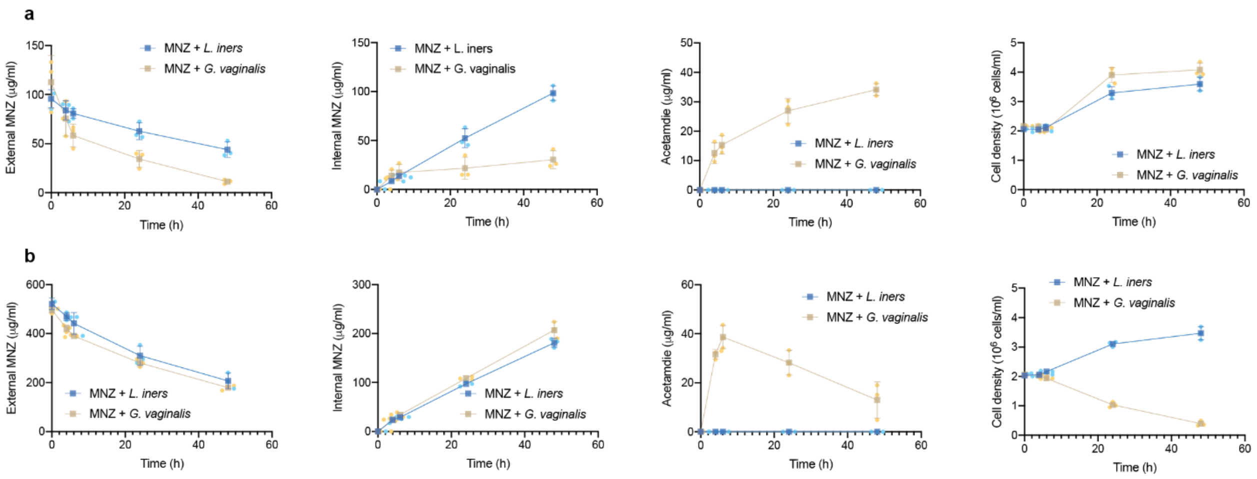

Supplementary Figure 1: Kinetic data for parameterization of MNZ interactions with *L. iners* and *G. vaginalis*. Kinetic data was collected at two doses of MNZ (a) low dose (100 µg/ml) and (b) high dose (500 µg/ml) for cultures with *Gv* and *Li* treated with MNZ Data are presented as mean ± SD, n = 3 biological replicates for each treatment group. Source data are provided as a Source Data file.

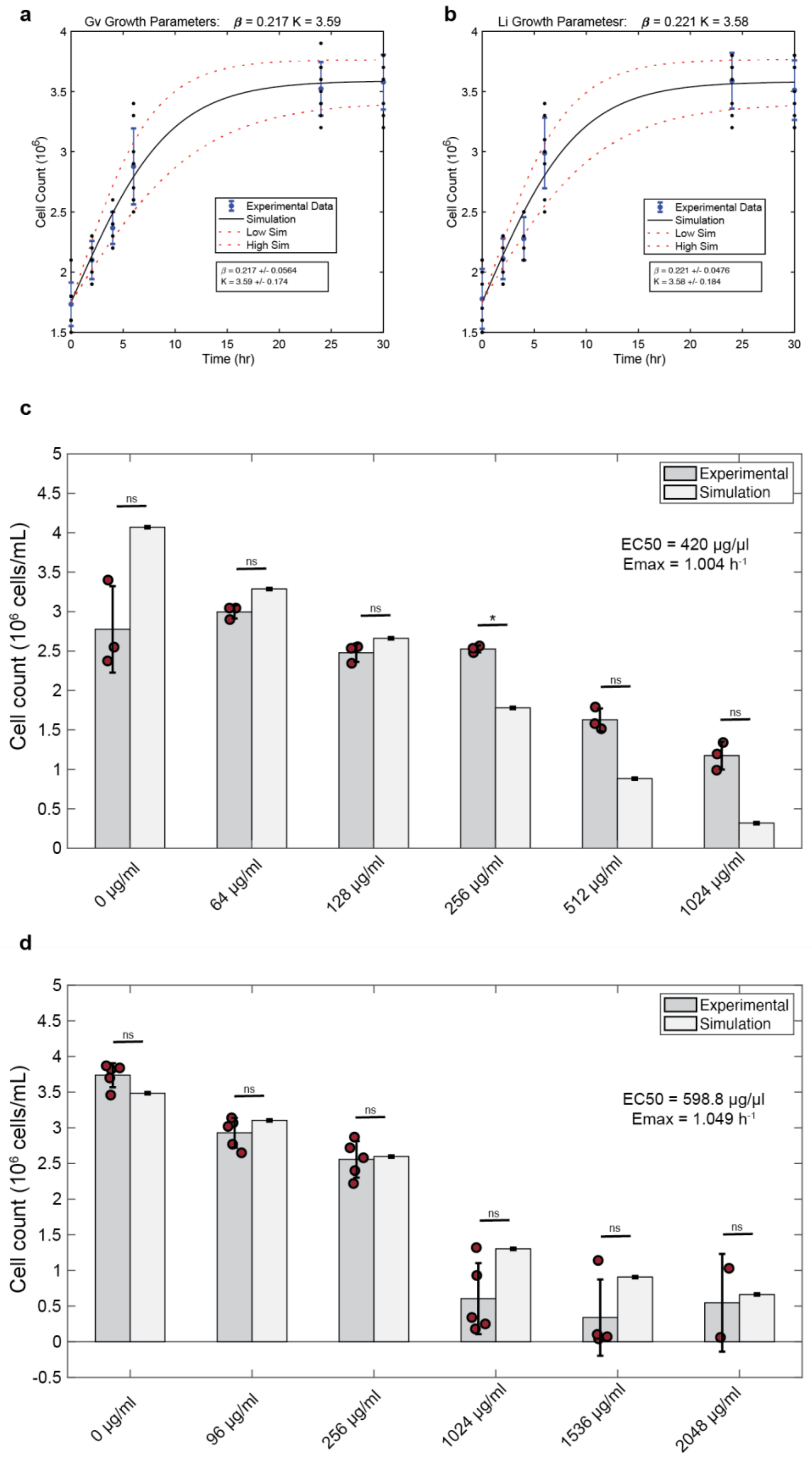

Supplementary Figure 2: Model parameterization of growth dynamics with and

without MNZ. a-b growth curves fit to a logistic equation using least-squares regression, growth rates and carrying capacity was determined from this data (n = 9 independent, biological replicates for each species). Red dashed lines represent confidence interval, values represent mean ± SD. **c-d** Kill curves used to determine the maximal kill rate and EC50 of MNZ for *Gv* (n = 3 independent, biological replicates for each dose, statistical analysis was completed using multiple unpaired two-tailed t-tests corrected using the Benjamini and Hochberg method: P = 0.212, t ratio = 2.05, df = 2; P = 0.152, t ratio = 2.90, df = 2; P = 0.415, t ratio = 1.02, df = 2; P = 0.030, t ratio = 14.2, df = 2; P = 0.104, t ratio = 4.58, df = 2; P = 0.104, t ratio = 4.21, df = 2) and *Li* (n = 5 independent, biological replicates for each dose, statistical analysis was completed using multiple unpaired two-tailed t-tests corrected using the Benjamini and Hochberg method: P = 0.736, t ratio = 1.37, df = 4; P = 0.736, t ratio = 0.758, df = 4; P = 0.912, t ratio = 0.135, df = 4; P = 0.736, t ratio = 1.282, df = 4; P = 0.736, t ratio = 0.950, df = 3; P = 0.912, t ratio = 0.1388, df = 1). Data in **c-d** are presented as mean ± SD, *P-value* *P < 0.05. Source data are provided as a Source Data file.

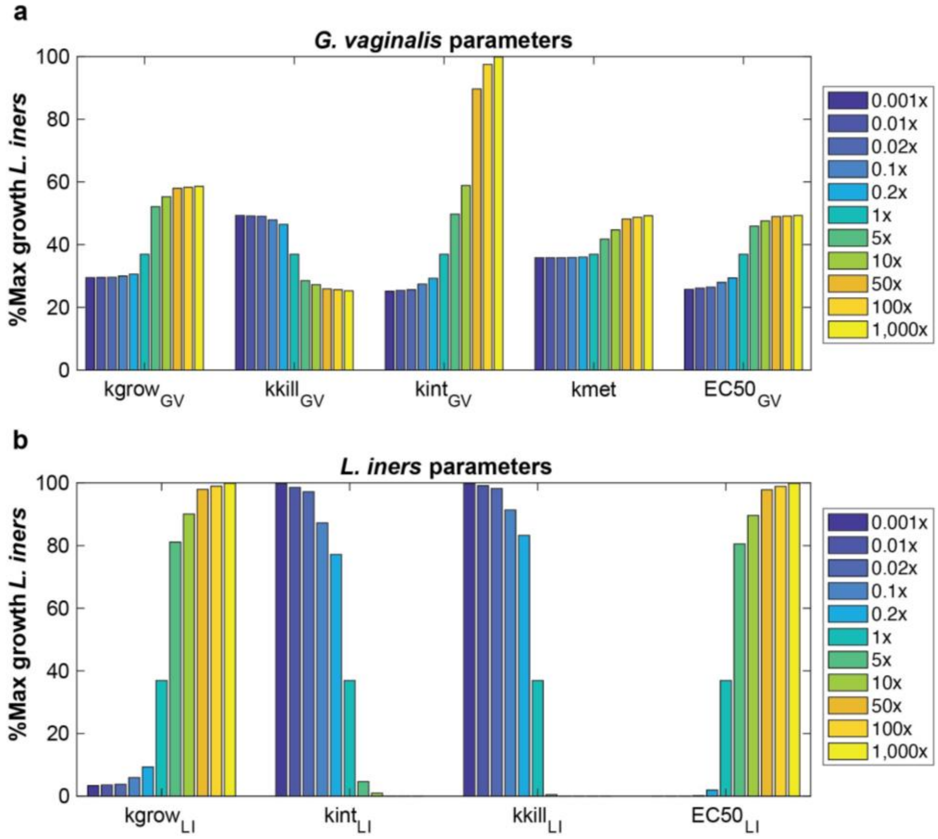

Supplementary Figure 3*: L. iners* 1D sensitivity analysis. a Sensitivity of *Li* growth with 500 μg/ml MNZ when parameters directly related to *Li* growth and survival are varied 0.001x to 1,000x fold baseline values. Percent maximal growth refers to the final cell count compared to the carrying capacity of the culture, the maximum cell density the culture can reach. **b** Sensitivity of *Li* growth with 500 μg/ml MNZ when parameters related to *Li* survival are varied 0.001x to 1,000x fold baseline values.

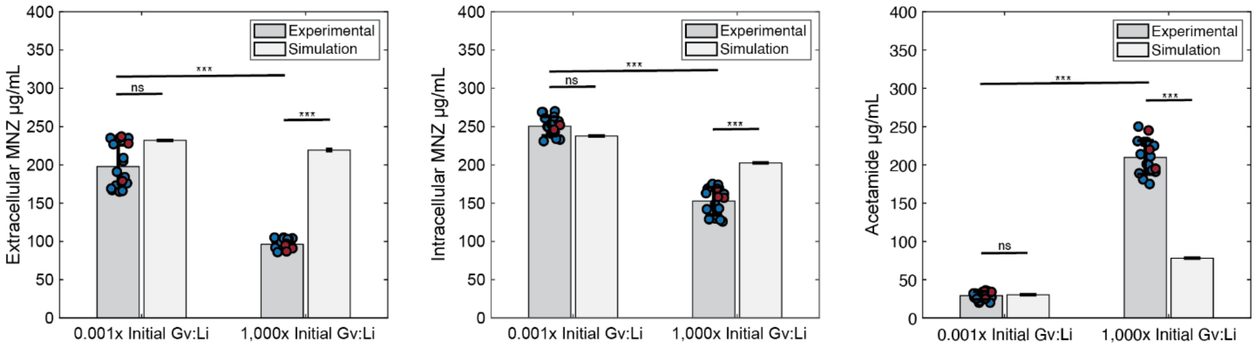

Supplementary Figure 4: Comparison of model predictions with observed levels of MNZ and metabolites. Validation for the model for extracellular MNZ, intracellular MNZ and acetamide. Intracellular MNZ is the sum of MNZ concentration in *Li* and *Gv* in the model. Statistical analyses were completed with unpaired two-sided t-tests. Extracellular MNZ 0.001x initial Gv:Li ratio and 1000x initial Gv:Li ratio experimental vs simulation, and experimental vs experimental P-values were: P = 0.2551, t = 1.178, df = 17; P = 2.17×10^{-12}, t = 17.70, df = 17; P = 2.33×10^{-16}, t = 14.77, df = 34, respectively. Intracellular MNZ: P = 0.3356, t = 0.991, df = 17; P = 0.0149, t = 2.71, df = 17; P = 1.12×10^{-19}, t = 18.99, df = 34. Acetamide: P = 0.8766, t = 0.1576, df = 17; P = 2.24×10^{-5}, t = 5.811, df = 17; P = 9.68×10^{-28}, t = 33.77, df = 34. *P-values:* * P < 0.05, ** P < 0.01, *** P < 0.001. Data are presented as mean ± SD with n = 18 biological replicates for each ratio. Source data are provided as a Source Data file.

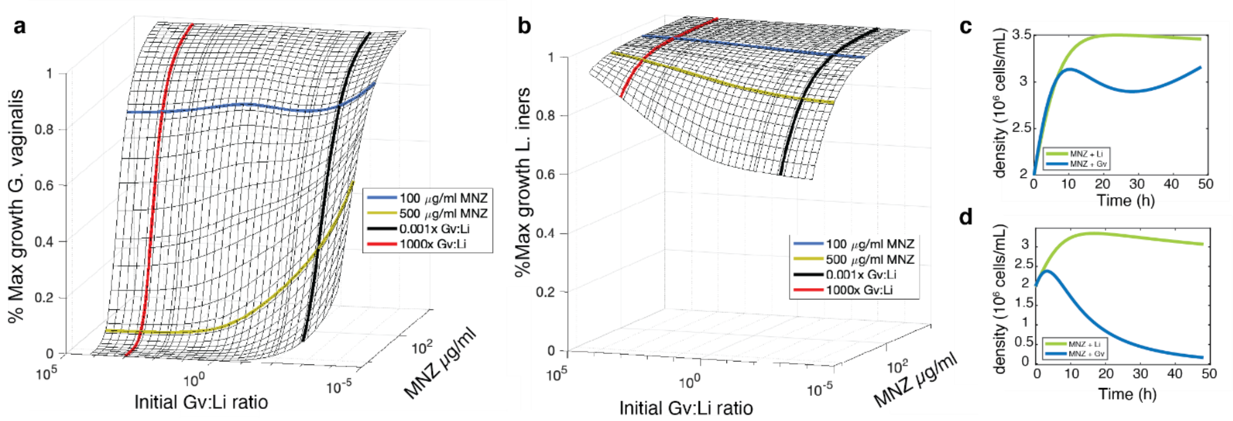

Supplementary Figure 5: *L. iners* susceptibility to MNZ does not influence Gv:Li ratio dependent MNZ efficacy. a – b Surface plot to illustrate predicted percent maximal growth of *Gv* and *Li* (z-axis) when concentration of MNZ (x-axis) and the initial ratio of Gv:Li (y-axis) are varied in simultaneously. **c – d** Model predicted growth dynamics for monoculture response to MNZ at 100 µg/ml and 500 µg/ml, respectively.

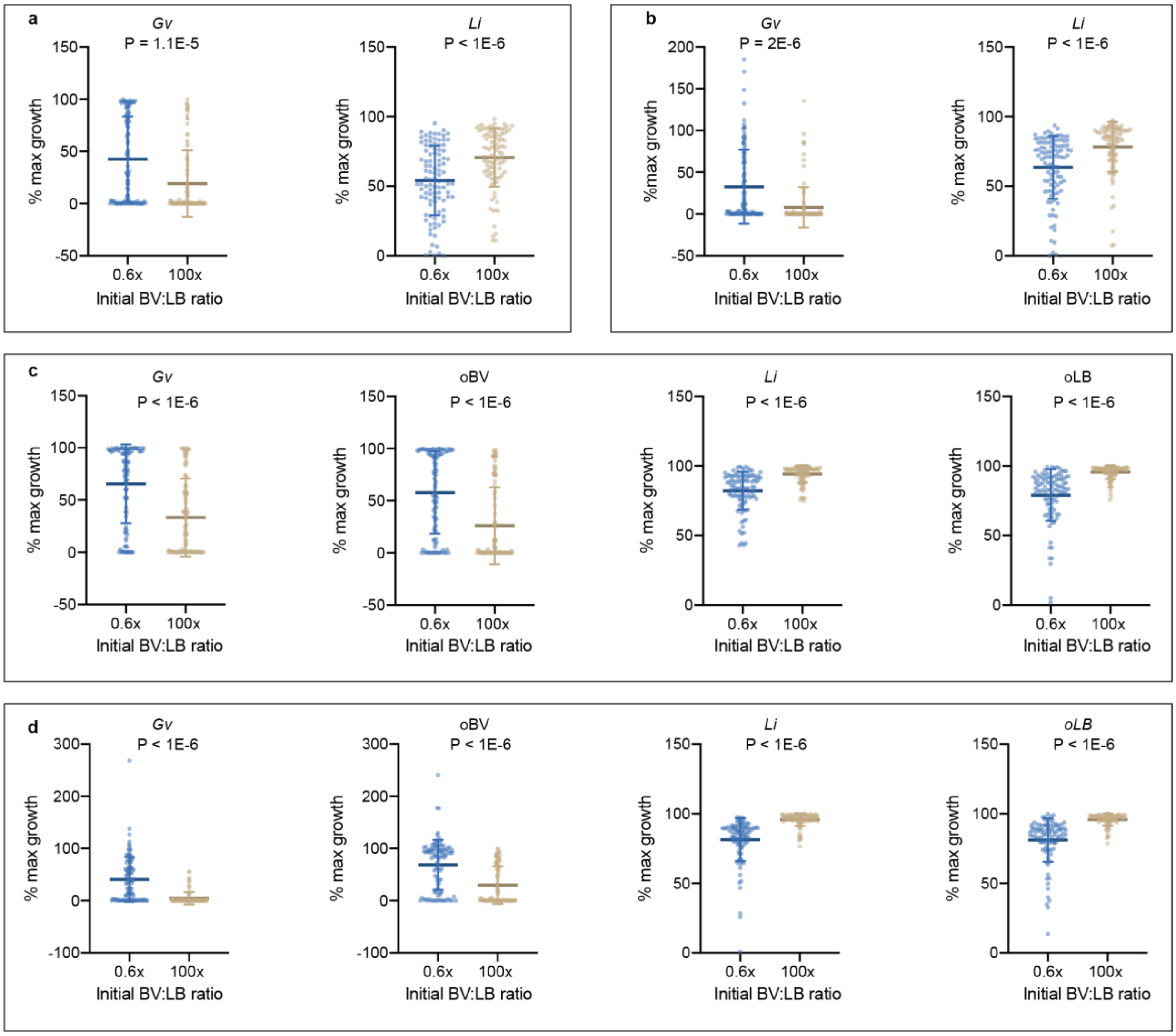

Supplementary Figure 6: BV:LB ratios influence endpoint BV-associated bacteria and *Lactobacillus* spp. abundances even with strain variation. a Model A: two species, no interactions percent of maximal growth for *Gv* (P = 1.08×10^{-5}, t ratio = 4.52, df = 198) and *Li* (P = 1.40×10^{-6}, t ratio = 5.06, df = 198). **b** Model B: two species with interactions percent of maximal growth for *Gv* (P = 2.21×10^{-6}, t ratio = 4.877, df = 198) and *Li* (P = 1.17×10^{-6}, t ratio = 5.10, df = 198). **c** Model C: four species, no interactions percent of maximal growth for *Gv* (P = 6.89×10^{-9}, t ratio = 6.10, df = 198), other BV-associated bacteria (oBV, P = 1.62×10^{-8}, t ratio = 5.89, df = 198), *Li* (P = 9.08×10^{-14}, t ratio

= 8.10, df = 198), and other *Lactobacillus* sp. (oLB, P = 6.50x10^{-15}, t ratio = 8.59, df = 198). **d** Model D: four species, with interactions percent of maximal growth for *Gv* (P = 1.03x10^{-13}, t ratio = 8.08, df = 198)*,* other BV-associated bacteria (oBV, P = 7.88x10^{-10}, t ratio = 6.46, df = 198), *Li* (P = 9.73x10^{-16}, t ratio = 8.89, df = 198), and other *Lactobacillus* sp. (oLB). Data are presented as mean ± SD, multiple unpaired two-tailed t-test p-values were adjusted using Benjamini and Hochberg correction, n = 100 independent simulations for each ratio.

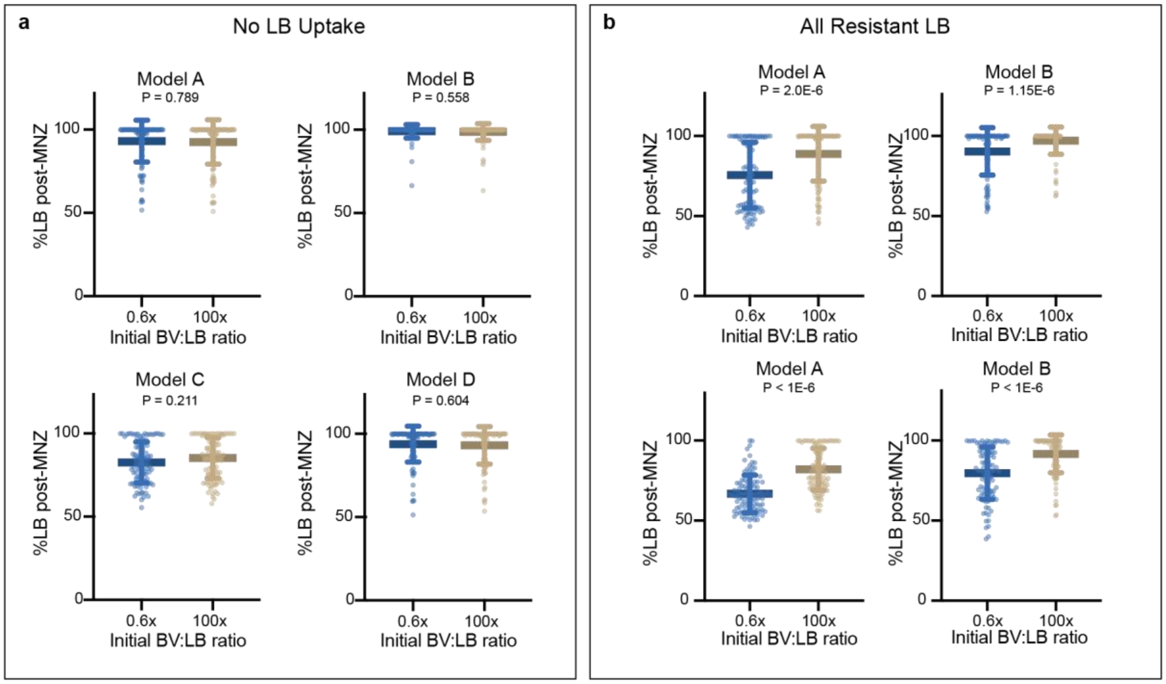

Supplementary Figure 7: Uptake or Sequestration of *Lactobacillus* spp. with MNZ drives initial BV:LB ratio influence of MNZ efficacy. a Post-treatment proportion of *Lactobacillus* sp. for Models A – D at 48h with 500 μg/ml MNZ with the rate of internalization of all *Lactobacillus* spp. (LB) set to zero. P-values for Model A-D respectively: P = 0.789, t ratio = 0.268, df = 198; P = 0.558, t ratio = 0.587, df = 198; P = 0.211, t ratio = 1.54, df = 198; P = 0.604, t ratio = 0.519, df = 198. **b** Post-treatment proportion of *Lactobacillus* sp. for Models A – D at 48h with 500 μg/ml MNZ with the EC50 set to 10,000 μg/ml for all *Lactobacillus* spp. (LB). P-values for Model A-D respectively: P = $2.04x10^{-6}$, t ratio = 4.98, df = 198; P = $1.15x10^{-4}$, t ratio = 3.94, df = 198; P = $3.68x10^{-15}$, t ratio = 8.68, df = 198; P = $2.35x10^{-8}$, t ratio = 6.00, df = 198. Data are presented as mean ± SD, multiple unpaired two-sided t-test p-values were adjusted using Benjamini and Hochberg correction, n = 100 independent simulations for each ratio. Source data are provided as a Source Data file.

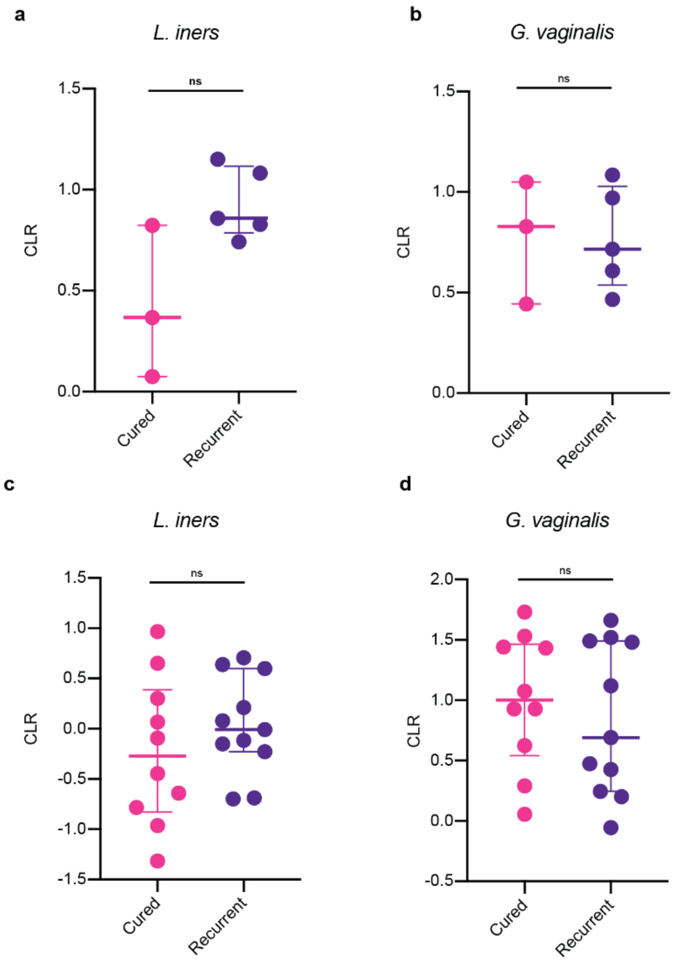

Supplementary Figure 8: Initial relative abundance data for *L. iners* and *G. vaginalis* for clinical outcomes. a - b UMB-HMP cohort (n = 3 individuals for cured group, n = 5 individuals for recurrent group) for CLR-transformed relative abundances of *L. iners* (P = 0.201, t = 2.69, df = 6) and *G. vaginalis* (P = 0.984, t = 0.0204, df = 6). **c - d** CONRAD BV cohort (n = 10 individuals for cured group, n = 11 individuals for recurrent group) CLR-transformed relative abundances for *L. iners* (P = 0.521, t = 0.963, df = 19) and *G. vaginalis* (P = 0.694, t = 0.624, df = 19). Data are presented as median (centre), 25th and 75th percentiles. Statistical analysis was completed with multiple two-sided unpaired t-tests, with p-values adjusted for multiple comparisons using the Benjamini and Hochberg method. Source data are provided as a Source Data file.

Supplementary Table 1: Parameters for two species model.

Parameter Name	Description	Value	Units	Ref
$k_{int\text{-}GV}$	internalization rate of MNZ into GV	0.0139	cell density^{-1} hr^{-1}	[1]
$k_{grow\text{-}GV}$	maximal growth rate of GV	0.2269	hr^{-1}	[2]
K_{GV}	carrying a capacity for GV	4.2	cell density (10^{6} mL^{-1})	[2]
$k_{kill\text{-}GV}$	kill rate of MNZ on GV	1.004	hr^{-1}	[3]
$EC50_{GV}$	concentration of MNZ to kill 50% of GV	420	µg mL^{-1}	[3]
k_{met}	rate of MNZ conversion to unknown metabolites	0.0174	cell density^{-1} hr^{-1}	[1]
$k_{int\text{-}LI}$	internalization rate of MNZ into LI	0.0042	cell density^{-1} hr^{-1}	[1]
$k_{grow\text{-}LI}$	maximal growth rate of LI	0.2309	hr^{-1}	[2]
K_{LI}	carrying a capacity for LI	3.569	cell density (10^{6} mL^{-1})	[2]
$k_{kill\text{-}LI}$	kill rate of MNZ on LI	1.049	hr^{-1}	[3]
$EC50_{LI}$	concentration of MNZ to kill 50% of LI	598.87	µg mL^{-1}	[3]

[1] Determined from fig. S1

[2] Determined from fig. S2a & b

[3] Determined from fig. S2c & d

Supplementary Table 2: Parameter ranges used to simulate intra-species variability.

	Units	*G. vaginalis*	Other BV-associated	*L. iners*	Other *Lactobacillus*
1) k_{int}	cell density^{-1}hr^{-1}	0.015 – 0.15	0.0 – 0.20	0.0015 – 0.15	0.0 – 0.10
2) k_{grow}	hr^{-1}	0.20 – 0.60	0.20 – 0.40	0.20 – 0.80	0.20 – 1.00
3) K	cell density (10^6 mL^{-1})	3.0 – 4.5	2.0 – 4.5	3.0 – 5.0	3.0 – 5.0
4) EC50	µg mL^{-1}	50 - 500	50 - 500	400 – 4,000	400 – 4,000
5) k_{kill}	hr^{-1}	1	1	1	1
6) k_{met}	cell density^{-1}hr^{-1}	0.005 – 0.05	0.005 – 0.05	0	0

Supplementary Table 3: Inter-species interaction terms. These terms descript the fold change in bacterial population that occurred from monoculture compared co-culture, a number greater than 1 indicates an increase in growth and less than 1 indicates an inhibition of growth.

		Source ($f_{s\text{->}t}$)			
		Gv	oBV	Li	oLB
Target	Gv		1.0 – 1.3	1.0 – 1.0	1x10^{-6} – 1.0
	oBV	1.0 – 2.5		1.0 – 1.0	1x10^{-6} – 1.0
	Li	1.0 – 1.0	1.0 – 1.0		1.0 – 1.3
	oLB	1.0 – 1.0	1.0 – 1.0	1.0 – 1.3	

Grey shading indicates self-interaction term, which we incorporated into the model in terms of carrying capacity and growth rate.

Supplementary Table 4: UMB-HMP cohort data.

Patient ID	log10 (BV:LB ratio)	BV:LB ratio	Total BV relative abundance	Total LB relative abundance	log10 (Gv:Li ratio)	Gv:Li ratio	*Li relative abundance*	*Gv relative abundance*
UAB128	0.291	1.954	0.620	0.317	-0.164	0.685	0.492	0.337
UAB003	0.185	1.532	0.574	0.374	-0.044	0.903	0.372	0.336
UAB005	0.090	1.230	0.517	0.420	-0.068	0.855	0.318	0.272
UAB035	0.086	1.219	0.502	0.411	-0.165	0.684	0.348	0.238
UAB053	0.104	1.269	0.535	0.422	-0.440	0.363	0.52	0.189
UAB127	0.961	9.143	0.799	0.087	0.683	4.817	0.093	0.448
UAB130	1.367	23.295	0.916	0.039	0.764	5.809	0.047	0.273
UAB135	0.192	1.555	0.548	0.352	-0.381	0.416	0.394	0.164

Grey shading denotes patients that exhibited recurrent BV.

Supplementary Table 5: CONRAD BV cohort data.

Patient ID	log10 (BV:LB ratio)	BV:LB ratio	Total BV relative abundance	Total LB relative abundance	log10 (Gv:Li Ratio)	Gv:Li ratio	*Gv relative abundance*	*Li relative abundance*
24_v1	0.976	9.453	0.713	0.075	0.667	4.640	0.348	0.075
25_v1	1.357	22.747	0.386	0.017	0.247	1.765	0.03	0.017
26_v1	0.577	3.776	0.609	0.161	-1.304	0.050	0.008	0.161
27_v1	1.537	34.442	0.136	0.004	0.628	4.250	0.017	0.004
28_v1	1.303	20.071	0.837	0.042	0.176	1.500	0.063	0.042
29_v1	1.722	52.692	0.290	0.006	0.544	3.500	0.021	0.006
30_v1	1.156	14.307	0.293	0.020	-0.125	0.750	0.015	0.02
31_v1	1.254	17.934	0.723	0.040	0.495	3.125	0.125	0.04
33_v1	1.465	29.206	0.581	0.020	0.860	7.250	0.145	0.02
34_v1	0.697	4.975	0.660	0.133	-0.391	0.406	0.054	0.133
35_v1	0.712	5.155	0.656	0.127	0.437	2.738	0.345	0.126
14_v1	1.579	37.958	0.824	0.022	0.740	5.500	0.121	0.022
15_v1	1.803	63.541	0.438	0.007	0.660	4.571	0.032	0.007
16_v1	2.369	233.627	0.823	0.004	1.224	16.750	0.067	0.004
17_v1	1.973	93.879	0.798	0.009	1.139	13.778	0.124	0.009
18_v1	0.896	7.868	0.747	0.095	0.282	1.916	0.182	0.095
19_v1	1.290	19.506	0.342	0.018	-0.051	0.889	0.016	0.018
20_v1	1.723	52.821	0.783	0.015	0.000	1.000	0.015	0.015
21_v1	2.032	107.535	0.223	0.002	1.230	17.000	0.034	0.002
22_v1	0.675	4.734	0.633	0.134	0.236	1.723	0.224	0.13
23_v1	2.578	378.471	0.440	0.001	1.114	13.000	0.013	0.001

Grey shading denotes patients that exhibited recurrent BV.

Supplementary Equations (Base Model Parameters)

$$\frac{d[\mathrm{Li}]}{dt} = [G_{Li} - D_{Li}] \cdot [\mathrm{Li}] \quad (1)$$

$$\frac{d[\mathrm{Gv}]}{dt} = [G_{Gv} - D_{Gv}] \cdot [\mathrm{Gv}] \quad (2)$$

$$\frac{d[\mathrm{MNZ_{ext}}]}{dt} = -k_{int\_Li} \cdot [\mathrm{MNZ_{ext}}] \cdot [\mathrm{Li}] - k_{int\_Gv} \cdot [\mathrm{MNZ_{ext}}] \cdot [\mathrm{Gv}] \quad (3)$$

$$\frac{d[\mathrm{MNZ_{int\_Li}}]}{dt} = k_{int\_Li} \cdot [\mathrm{MNZ_{ext}}] \cdot [Li] - D_{Li} \cdot [\mathrm{Li}] \cdot \left[\mathrm{MNZ}_{\frac{\mathrm{int}}{\mathrm{Li}}}\right] \quad (4)$$

$$\frac{d[\mathrm{MNZ_{int\_Gv}}]}{dt} = k_{int\_Gv} \cdot [\mathrm{MNZ_{ext}}] \cdot [\mathrm{Gv}] - k_{met} \cdot \left[\mathrm{MNZ_{int_{Gv}}}\right] \cdot [Gv] - D_{Gv} \cdot [\mathrm{Gv}] \cdot [\mathrm{MNZ}_{\frac{\mathrm{int}}{\mathrm{Gv}}}] \quad (5)$$

$$\frac{d[\mathrm{Met}]}{dt} = k_{met} \cdot \left[\mathrm{MNZ_{int_{Gv}}}\right] \cdot [\mathrm{Gv}] \quad (6)$$

$$D = k_{kill}\left(\frac{[\mathrm{MNZ_{int}}]}{\mathrm{EC50} + [\mathrm{MNZ_{int}}]}\right) \quad (7)$$

$$G = k_{grow}\left(1 - \frac{[\text{cell density}]}{K}\right) \quad (8)$$

$$\mathrm{MNZ_{int/cell}} = \frac{[\mathrm{MNZ_{int}}]}{[\text{cell density}] \cdot V_{culture}} V_{cell} \quad (9)$$

Equation (1) represents the growth dynamics of the *Li* population, Equation (2) the *Gv* population, Equation (3) the extracellular MNZ concentration, Equation (4) the bulk intracellular MNZ concentration for *Li,* Equation (5) the bulk intracellular MNZ concentration for *Gv,* Equation (6) the concentration of metabolites produced by *Gv*. In Equation (7), “D” represents the death rate of the population and is dependent on the respective intracellular MNZ concentrations, EC50s and maximum kill rates. In Equation (8), “G” represents the logistic growth of the respective populations dependent on the maximum growth rate and carrying capacities. In Equation (9), “$\mathrm{MNZ_{int/cell}}$” represents the mass of MNZ internalized in each cell and is dependent on the bulk concentration of MNZ, per cell (cell density * culture volume) and cell volume.
