# Supplementary material for: Quantitative modeling predicts mechanistic links between pre-treatment microbiome composition and metronidazole efficacy in bacterial vaginosis
Source: Nat Commun. 2020 Dec 1;11:6147. doi: 10.1038/s41467-020-19880-w (PMC7708644; doi:10.1038/s41467-020-19880-w)
Supplement: Supplementary file 3 — Reporting Summary [file 41467_2020_19880_MOESM3_ESM.pdf]

nature research

Corresponding author(s): Klatt, Arnold

Last updated by author(s): Oct 21, 2020

Reporting Summary

Nature Research wishes to improve the reproducibility of the work that we publish. This form provides structure for consistency and transparency in reporting. For further information on Nature Research policies, see our Editorial Policies and the Editorial Policy Checklist.

Statistics

For all statistical analyses, confirm that the following items are present in the figure legend, table legend, main text, or Methods section.

n/a	Confirmed	
☐	☒	The exact sample size (n) for each experimental group/condition, given as a discrete number and unit of measurement
☐	☒	A statement on whether measurements were taken from distinct samples or whether the same sample was measured repeatedly
☐	☒	The statistical test(s) used AND whether they are one- or two-sided<br>*Only common tests should be described solely by name; describe more complex techniques in the Methods section.*
☒	☐	A description of all covariates tested
☒	☐	A description of any assumptions or corrections, such as tests of normality and adjustment for multiple comparisons
☐	☒	A full description of the statistical parameters including central tendency (e.g. means) or other basic estimates (e.g. regression coefficient) AND variation (e.g. standard deviation) or associated estimates of uncertainty (e.g. confidence intervals)
☐	☒	For null hypothesis testing, the test statistic (e.g. F, t, r) with confidence intervals, effect sizes, degrees of freedom and P value noted<br>*Give P values as exact values whenever suitable.*
☒	☐	For Bayesian analysis, information on the choice of priors and Markov chain Monte Carlo settings
☒	☐	For hierarchical and complex designs, identification of the appropriate level for tests and full reporting of outcomes
☒	☐	Estimates of effect sizes (e.g. Cohen's d, Pearson's r), indicating how they were calculated

Our web collection on statistics for biologists contains articles on many of the points above.

Software and code

Policy information about availability of computer code

Data collection	No software was used for data collection.
Data analysis	GraphPad Prism 8 was used for the statistical analysis and Matlab 2018b was used to generate custom code that was central to the research and not yet described in published literature. Matlab 2018 was also used to get exact p-values, when GraphPad Prism 8 could not calculate them. The code is deposited and Github and can be accessed at https://doi.org/10.5281/zenodo.4121904 .

For manuscripts utilizing custom algorithms or software that are central to the research but not yet described in published literature, software must be made available to editors and reviewers. We strongly encourage code deposition in a community repository (e.g. GitHub). See the Nature Research guidelines for submitting code & software for further information.

Data

Policy information about availability of data

All manuscripts must include a data availability statement. This statement should provide the following information, where applicable:

- Accession codes, unique identifiers, or web links for publicly available datasets
- A list of figures that have associated raw data
- A description of any restrictions on data availability

The source data are provided with this paper for Figures 2-5, Supplementary Figure 1-2, 4 and 8, which includes the in vitro validation, model parameterization and clinical validation. For the clinical studies, the UMB-HMP cohort study sequence data and metadata were deposited in the Sequence Read Archive (SRA; http://www.ncbi.nlm.nih.gov/Traces/sra/) under BioProject PRJNA208535 ("The daily dynamics of the vaginal microbiota before and after bacterial vaginosis"; http://www.ncbi.nlm.nih.gov/bioproject/? term=PRJNA208535) ([SRP026107] and [SRA091234]).33 An abbreviated data set necessary for the reproduction of Fig. 5a-b and Supplementary Fig. 8a-b are in Supplementary Table 4 and the Source Data file. The sequence data and metadata for the CONRAD BV study are not in a formal repository, but are fully available upon request; however, we have included an abbreviated version of this data set that includes all the data necessary for

reproduction of Fig. 5c-d and Supplementary Fig. 8c-d are in Supplementary Table 5 and can be found in the Source Data file.

Field-specific reporting

Please select the one below that is the best fit for your research. If you are not sure, read the appropriate sections before making your selection.

[x] Life sciences [ ] Behavioural & social sciences [ ] Ecological, evolutionary & environmental sciences

For a reference copy of the document with all sections, see nature.com/documents/nr-reporting-summary-flat.pdf

Life sciences study design

All studies must disclose on these points even when the disclosure is negative.

Sample size	The sample sizes for the experiments were not pre-determined statistically. The sample size of the in vitro monoculture data used for model parameterization which ranged from n = 3 for the parameterization of MNZ uptake and metabolism, n = 9 for the growth rates and carrying capacity, and n = 3-5 for sensitivity of the bacteria to MNZ. Completing these experiments in triplicate is typical in the field (Atassi, F., et al. Diverse Expression of Antimicrobial Activities Against Bacterial Vaginosis and Urinary Tract Infection Pathogens by Cervicovaginal Microbiota Strains of Lactobacillus gasseri and Lactobacillus crispatus. Front. Microbiol. 10, (2019); Jackman, C. M., et al. Microdroplet co-cultivation and interaction characterization of human vaginal bacteria. Int Bio (Cam) 11, 69–78 (2019)). As accuracy in parameterization was not the central goal of this manuscript the use of 3-9 independent biological replicates was satisfactory, and we additionally completed simulations over ranges of possible parameter values observed in the literature (Fig. 4, Supplementary Tables 2-3). Moreover, since we later validated the model that were based on measurements from monoculture data in co-culture, we further supported that the sample sizes and estimations from the monoculture were sufficient. We selected a larger sample size for the in vitro validation study (n = 18) based on previous experiments in the Klatt Lab between vaginal microbiota and drugs (Klatt, N. R. et al. Vaginal bacteria modify HIV tenofovir microbicide efficacy in African women. Science 356, 938–945 (2017)). The de-identified clinical data sample size was limited to data previously collected in those studies.
Data exclusions	Exclusion criteria were not pre-established for the in vitro data, but no data was excluded from in vitro experimental measurements. Data was excluded from the clinical cohorts based on the following predetermined (in regards to our statistical analysis) criteria (stated in the Methods text): 1) MNZ regimen was not completed; 2) Individual did not have BV according to Nugent scoring at the time of MNZ treatment; 3) Individual did not have follow-up data available; 4) Individual did not exhibit treatment failure as defined in the manuscript (resolved BV at an intermediate time point followed by a positive test for BV).
Replication	To verify the trends observed in the clinical data, we looked at two independent studies in distinct study populations. The in vitro data had a sample size of n = 18 co-cultures for each test condition. Additionally, when we observed variability in L. iners growth, we completed simulations to determine how growth dynamic variability influenced the model findings. All attempts at replication of model findings were successful.
Randomization	The in vitro bacterial mono and co-cultures were not randomized, and no covariates are anticipated to influence the results as the experiments were all completed by the same individual and same setting. Clinical data was randomized as previously described in their respective publications (Ravel et al., 2013 (PMID: 24451163) for the UMB-HMP data and Thurman et al., 2015 (PMID: 26204200) for the BV Conrad data).
Blinding	Computational prediction of model findings by CYL was initially blinded to the experimental validation results by RKC, until experimenter confirmed the ratio dependent trends were observed in the data. Clinical data was blinded as previously described in their respective publications (Ravel et al., 2013 (PMID: 24451163) for the UMB-HMP data and Thurman et al., 2015 (PMID: 26204200) for the BV Conrad data).

Reporting for specific materials, systems and methods

We require information from authors about some types of materials, experimental systems and methods used in many studies. Here, indicate whether each material, system or method listed is relevant to your study. If you are not sure if a list item applies to your research, read the appropriate section before selecting a response.

Materials & experimental systems

n/a	Involved in the study	
[x]	[ ]	Antibodies
[x]	[ ]	Eukaryotic cell lines
[x]	[ ]	Palaeontology and archaeology
[x]	[ ]	Animals and other organisms
[x]	[ ]	Human research participants
[ ]	[x]	Clinical data
[x]	[ ]	Dual use research of concern

Methods

n/a	Involved in the study	
[x]	[ ]	ChIP-seq
[x]	[ ]	Flow cytometry
[x]	[ ]	MRI-based neuroimaging

Clinical data

Policy information about clinical studies

All manuscripts should comply with the ICMJEguidelines for publication of clinical research and a completedCONSORT checklist must be included with all submissions.

Clinical trial registration	We used de-identified data from the Bacterial Vaginosis CONRAD study published in Thurman et al., 2015 (PMID: 26204200). The Thurman et al., 2015 data was registered with ClinicalTrials.gov (#NCT01347632). We also used de-identified data from the University of Maryland Baltimore Human Microbiome Project published in Ravel et al., 2013 (PMID: 24451163). The University of Maryland Baltimore Human Microbiome Project was an observational study, not a clinical trial and all detailsl for the original study are include din Ravel et al., 2013 (PMID: 24451163), which was approved by institutional review boards at the University of Alabama and the University of Maryland.
Study protocol	Study protocol can be obtained from original publications: Ravel et al., 2013 (PMID: 24451163) and Thurman et al., 2015 (PMID: 26204200).
Data collection	Extended data collection information can be obtained from original publications: Ravel et al., 2013 (PMID: 24451163) and Thurman et al., 2015 (PMID: 26204200). Briefly, the UMB-HMP cohort was an observational study which consisted of a total of 135 non-pregnant women of reproductive age at the University of Alabama at Birmingham over the course of 11 weeks who were enrolled in the longitudinal study between September 2009 and July 2010. The CONRAD BV cohort was collected at the Eastern Virginia Medical School for 69 non-pregnant reproductive aged women between April 2011 and December 2011.
Outcomes	Clinical outcomes can be obtained from original publications: Ravel et al., 2013 (PMID: 24451163) and Thurman et al., 2015 (PMID: 26204200). The UMB-HMP cohort was not a clinical trial, but rather an observational prospective study with no intervention. The goal of the UMP-HMP study was to evaluate how vaginal microbial communities vary prior to, during and after episodes of BV by characterizing the composition and dynamics of vaginal bacterial communities. The primary study outcome for the CONRAD BV study was p24 antigen production in tissue and evaluate HIV infection and safety of cervico-vaginal tissue in women during BV infection, 1 week after 7-day course of metronidazole therapy and 1 month after metronidazole therapy. The secondary outcome was to interrogate enhancement of pro-inflammatory response during BV and BV treatment as well as measure un-culturable bacteria by 16S RNA qPCR. Since our analysis was independent of the initial goals, primary or secondary outcomes of these studies, we do not have primary and secondary outcome measures to define for this study.
